# Systemic Analysis of the DNA Replication Regulator MCM Complex in Ovarian Cancer and Its Prognostic Value

**DOI:** 10.3389/fonc.2021.681261

**Published:** 2021-06-09

**Authors:** Yukun Li, Juan Zou, Qunfeng Zhang, Feifei Quan, Lu Cao, Xiaodi Zhang, Jue Liu, Daichao Wu

**Affiliations:** ^1^ Department of Obstetrics and Gynecology, The Second Affiliated Hospital of University of South China, Hengyang, China; ^2^ Department of Obstetrics and Gynecology, Foshan First People’s Hospital, Foshan, China; ^3^ Clinical Anatomy & Reproductive Medicine Application Institute, Department of Histology and Embryology, University of South China, Hengyang, China; ^4^ Institute for Bioscience and Biotechnology Research, University of Maryland, Rockville, MD, United States

**Keywords:** ovarian cancer, The minichromosome maintenance (MCM) complex, public databases, prognostic value, comprehensive bioinformatics

## Abstract

Microliposome maintenance (MCM) 2, MCM3, MCM4, MCM5, MCM6, and MCM7 are DNA replication regulators and are involved in the progression of multiple cancer types, but their role in ovarian cancer is still unclear. The purpose of this study is to clarify the biological function and prognostic value of the MCM complex in ovarian cancer (OS) progression. We analyzed DNA alterations, mRNA and protein levels, protein structure, PPI network, functional enrichment, and prognostic value in OC based on the Oncomine, cBioPortal, TCGA, CPTAC, PDB, GeneMANIA, DAVID, KEGG, and GSCALite databases. The results indicated that the protein levels of these DNA replication regulators were increased significantly. Moreover, survival analysis showed a prognostic signature based on the MCM complex, which performed moderately well in terms of OS prognostic prediction. Additionally, protein structure, functional enrichment, and PPI network analyses indicated that the MCM complex synergistically promoted OC progression by accelerating DNA replication and the cell cycle. In conclusion, our study suggested that the MCM complex might be a potential target and prognostic marker for OC patients.

## Introduction

Ovarian cancer (OC) is a severe malignant disease of the female reproductive system, and it ranked eighth in terms of morbidity and mortality overall in 2018 (1). However, this malignant disease remains the main cause of death from gynecological cancer; the 5-year survival rate of advanced OC is less than 40% (2). For most OC patients, the initial response rate to the first treatment is excellent, but more than 60% of OC patients will have a recurrence within approximately eighteen months. After approximately 3 years, all OC patients will have a recurrence. When this occurs, this malignant disease is incurable (3). Therefore, it is very important to identify novel prognostic marks for OC patients, which may help distinguish OC patients at high risk, predict treatment prognosis and outcome, and even provide new therapeutic options.

The microliposome maintenance (MCM) complex is a group of six structurally related proteins that can interact to form a hexamer (4). The MCM complex can directly regulate the DNA replication system. Dysregulation of the MCM complex induces many human cancers (4, 5). Each regulator has been identified in different cancer types. For example, many studies have indicated that the expression of MCM2 is increased in oral squamous cell carcinoma (6), cervical carcinoma (7), and medulloblastoma (8). MCM3 is highly expressed in osteosarcoma (9), salivary gland tumors (10), and glioma (11). MCM4 and MCM7 are regarded as better biomarkers than Ki-67, Bmi1, and cyclin E for esophageal adenocarcinoma and precancerous lesions (12). A high level of MCM5 has been found in multiple cancers, including colon cancer (13), cervical cancer (14), and thyroid cancer (15). Ectopic expression of MCM6 has been found in liver cancer (16), endometrioid endometrial adenocarcinoma (17), and glioma (18). Nevertheless, there are few works in the literature on the biological function and prognostic value of the MCM complex for OC progression.

The aim of this study is to remedy this problem. We used the Oncomine, The Cancer Genome Atlas (TCGA), Clinical Proteomics Tumour Analysis Consortium (CPTAC), and Human Protein Atlas (HPA) databases to identify the mRNA and protein levels and prognostic value of the MCM complex in OC. Then, we confirmed the altered level of the MCM complex by the cBioPortal database and analyzed the structures of MCM2/3/4/5/6/7. Subsequently, we constructed a PPI network based on the GeneMANIA database. Furthermore, we predicted the Gene Ontology functions and pathways of the MCM complex and 20 relevant genes by the DAVID and KEGG databases. Ultimately, we also investigated the relationship between the MCM complex and immune infiltration based on the TIMER database. The present study analyzed the expression, potential functions, and prognostic values of the MCM complex in OC.

## Materials and Methods

### Different mRNA Levels of the MCM Complex in Public Databases

The ‘Oncomine database (https://www.oncomine.org)’ website is user-friendly (19). We analyzed the mRNA level of the MCM complex in multiple cancer types based on this database. The publicly available TCGA (https://www.cancer.gov/tcga) contains clinical, genomic variation, mRNA expression, and methylation level expression data for various human cancers (20). As a user‐friendly tool, GEPIA (http://gepia.cancer-pku.cn/) was used in the present study to analyze cancer transcriptome data (21). These public databases were used to analyze the transcriptional level of the MCM complex in OC.

### Differential Expression of MCM Proteins in the Databases

The CPTAC database (https://proteomics.cancer.gov/programs/cptac) integrates genomic and proteomic data to identify and characterize all proteins in tumor and normal tissues and to identify candidate proteins that can be used as tumor biomarkers (22). Moreover, differential expression analysis was performed using the UALCAN database (http://ualcan.path.uab.edu/index.html), which is an online tool for performing gene expression profiling analyses in cancer and adjacent tissues based on the CPTAC database and TCGA (23). The HPA (http://www.proteinatlas.org) database is a website for assessing protein levels in many cancer types and normal tissues based on an immunohistochemistry platform (24). These public databases were utilized to confirm the post-transcriptional and phosphorylation levels of the MCM complex in OC.

### DNA Alteration Analysis of MCM2-7

The relationship between MCM complex alterations and survival outcome in OC patients was analyzed by the cBioProtal database (http://www.cbioportal.org/) (25). We used this website to analyze DNA alterations of the MCM complex in OC, including the DNA alteration frequency and perform OS analysis of OC patients with or without MCM alterations.

### Protein Structure Analysis of MCMs

The Protein Data Bank (PDB) (https://www.rcsb.org/) was established as the 1st open access digital data resource in all biology and medicine (26). We utilized this database to analyze the secondary and tertiary structures of proteins in the MCM complex.

### MCM Complex Network Construction

GeneMANIA 3.6.0 (http://www.genemania.org) is an online tool for constructing PPI networks by using proteomics and genomics data (27). We utilized this web tool to generate a network according for the MCM complex. The maximum resultant attributes and genes were 10 and 20, respectively. These genes were used in subsequent analyses.

### GO and KEGG Enrichment Analyses

The DAVID database (https://david.ncifcrf.gov/) is a database of biological information and is available free online (28). At present, the DAVID database is mainly used to perform functional and pathway enrichment analyses of differential genes and is a very good tool used by many researchers. We submitted the 20 identified genes to the DAVID database and performed GO function and KEGG pathway analyses.

### Immune Infiltration Analysis of the MCM Complex

TIMER (https://cistrome.shinyapps.io/timer/) is a classical and authoritative database for analyzing immune infiltration in multiple cancer types (29). To understand the effect of the MCM complex on the immune infiltration of OC, the correlation between gene expression and the abundance of immune infiltrates was analyzed in the gene module, and the correlation between somatic CNAs and the abundance of immune infiltrates was analyzed in the SCNA module.

### Survival Analysis

Survival analysis of the MCM2, MCM3, MCM4, MCM5, MCM6, and MCM7 signatures and the six-gene MCM signature in OC patients was performed using the GEPIA database (http://gepia.cancer-pku.cn/) (21).

### Cell Culture and Transfection

Human ovarian cancer cell lines, A2780 (American Type Culture Collection), were cultured in RPMI−1640 medium (Sigma−Aldrich; Thermo Fisher Scientific, Inc.) supplemented with 1% (v/v) Penicillin–Streptomycin mixture (Thermo Fisher Scientific, Inc.) and 10% (v/v) fetal bovine serum (FBS; Gibco; Invitrogen; Thermo Fisher Scientific, Inc.). The hsa-miR-34a-5p and hsa-miR-23b-3p mimics were synthesized by Genepharma (Genepharma, Shanghai, China). Cells were transiently transfected with hsa-miR-34a-5p or hsa-miR-23b-3p mimics for 48 h using Lipofectamine 2000 (Invitrogen, Carlsbad, USA).

### Reverse Transcription−Quantitative PCR

qPCR was conducted as previously described (30). Primers used were listed as follows: GAPDH forward GTCTCCTCTGACTTCAACAGCG, GAPDH reverse: ACCACCCTGTTGCTGTAGCCAA; MCM2 forward: TGCCAGCATTGCTCCTTCCATC, MCM2 reverse: AAACTGCGACTTCGCTGTGCCA; MCM3 forward: CGAGACCTAGAAAATGGCAGCC, MCM3 reverse: GCAGTGCAAAGCACATACCGCA; MCM4 forward: CTTGCTTCAGCCTTGGCTCCAA, MCM4 reverse: GTCGCCACACAGCAAGATGTTG; MCM5 forward: GACTTACTCGCCGAGGAGACAT, MCM5 reverse: TGCTGCCTTTCCCAGACGTGTA; MCM6 forward: GACAACAGGAGAAGGGACCTCT, MCM6 reverse: GGACGCTTTACCACTGGTGTAG; and MCM7 forward: GCCAAGTCTCAGCTCCTGTCAT, MCM7 reverse: CCTCTAAGGTCAGTTCTCCACTC. miR-34a-5p forward: AGGGGGTGGCAGTGTCTTAG, reverse: GTGCGTGTCGTGGAGTCG. miR-23b-3p forward: GAGCATCACATTGCCAGGG, reverse: GTGCAGGGTCCGAGGT. U6 forward: CTCGCTTCGGCAGCACATA, reverse: AACGATTCACGAATTTGCGTC.

### Statistical Analysis

Statistical analyses were performed in the R Programming Language (version 3.6). All statistical tests were bilateral, and P <0:05 was statistically significant.

## Results

### The Transcriptional and Protein Levels of the MCM Complex in OC

**Figure 1:**
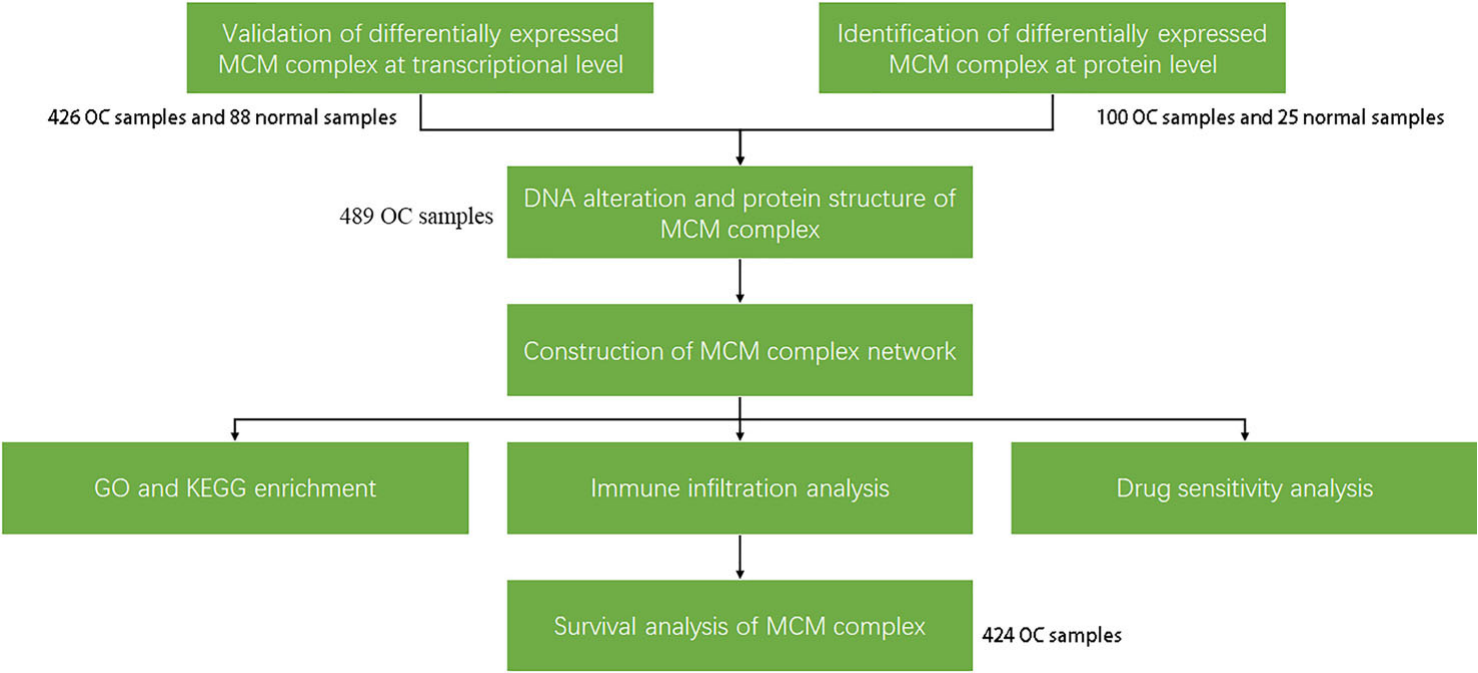
Work flow of the study. First, the transcriptional and post-transcriptional levels of the members of the MCM complex were confirmed by the Oncomine, GEPIA, UALCAN, CPTAC, and cBioPortal databases (p < 0.05 and |FDR| ≥2). There were differences at the transcriptional and post-transcriptional levels among the MCMs. Therefore, the post-transcriptional regulation (miRNA network and protein modification) among the MCM proteins was confirmed by the GSCALite and PDB databases. Then, we constructed the MCM complex network using the GeneMANIA database, which was used to predict GO functions and KEGG pathways. Immune infiltration and drug sensitivity analyses of the MCM complex were performed using the TIMER and GSCALite databases. Finally, we confirmed the effect of the MCM complex on survival based on the GEPIA database.

**Figure 2:**
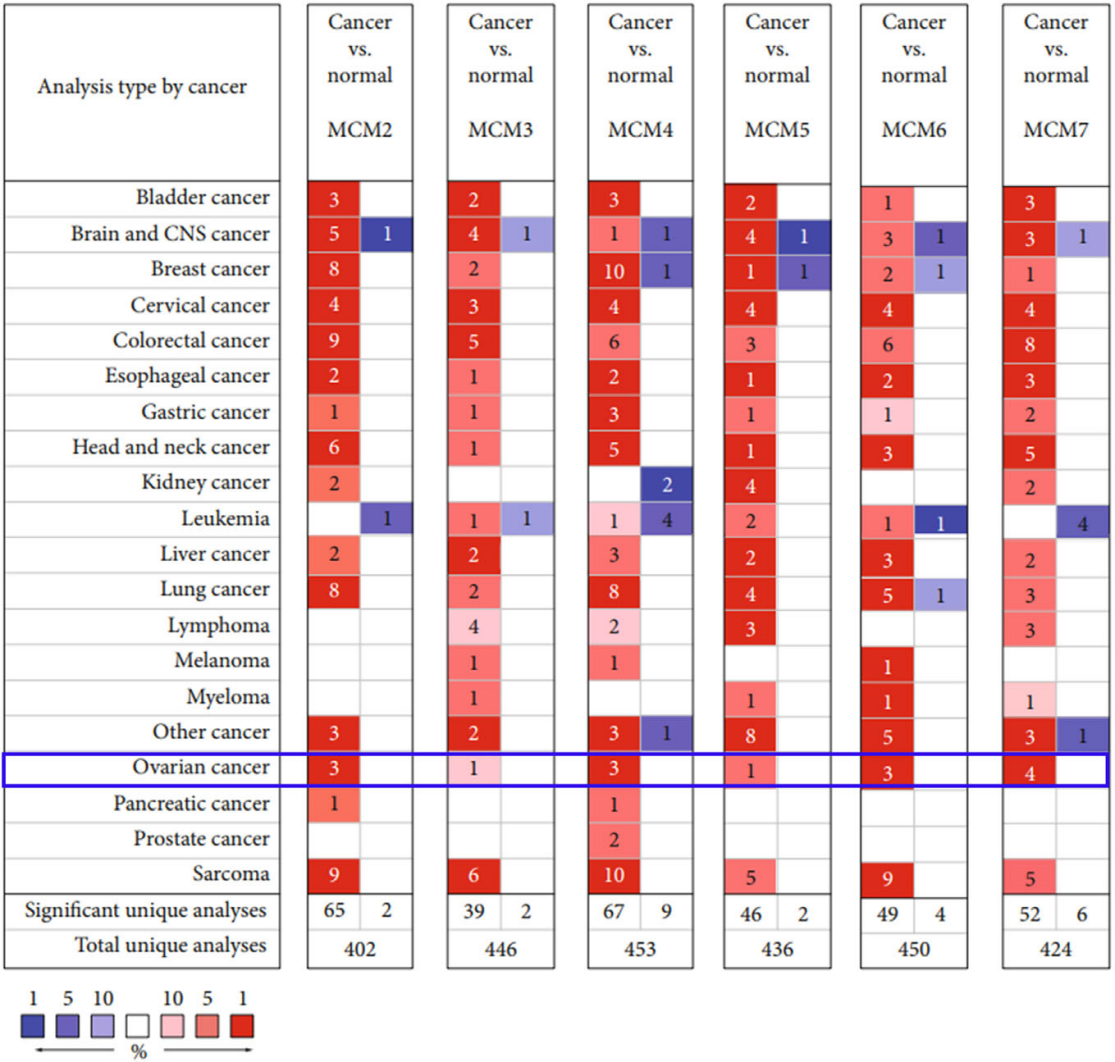
Transcriptional level of MCM complex members in 20 cancer types. The levels of the MCMs in different cancer types. The threshold (P value ≤ 0.05; ∣FDR ∣ ≥2; gene rank ≤ 10%; data type: mRNA) is expressed in colored cells. Red indicates that the gene is increased in cancer tissues compared to that in normal tissues, whereas blue indicates that the gene is downregulated in cancer tissues compared to that in normal tissues. The FDR is presented by color depth.

The research strategy is presented in **Figure 1**. The Oncomine database (www.ocomine.org) was used to identify the transcriptional level of the MCM complex in different cancer and corresponding para-carcinoma tissues (**Figure 2**).

We found that the MCM complex was enhanced in most cancer types, including bladder cancer, breast cancer, cervical cancer, colorectal cancer, esophageal cancer, gastric cancer, head and neck cancer, liver cancer, lung cancer, ovarian cancer, and sarcoma. In the total unique analyses, we found 402 datasets for MCM2, 446 datasets for MCM3, 453 datasets for MCM4, 436 datasets for MCM5, 450 datasets for MCM6, and 424 datasets for MCM7. Moreover, the expression of MCM2 in cancer tissue was significantly increased in 65 datasets and decreased in two datasets. Cancerous MCM3 expression was significantly enhanced in 39 datasets and reduced in two datasets compared to that in normal tissues. The levels of MCM4 in cancer were markedly upregulated in 67 datasets and downregulated in nine datasets compared to that in normal tissues. The MCM5 level was higher in 46 cancer datasets and lower in two cancer datasets than in normal tissue datasets. High expression of MCM6 was found in 49 cancer datasets compared to that in normal tissue datasets. Low expression of MCM6 was found in four datasets. The MCM7 level was also increased in 52 cancer datasets and decreased in six cancer datasets compared to that in normal tissue datasets.

**Figure 3:**
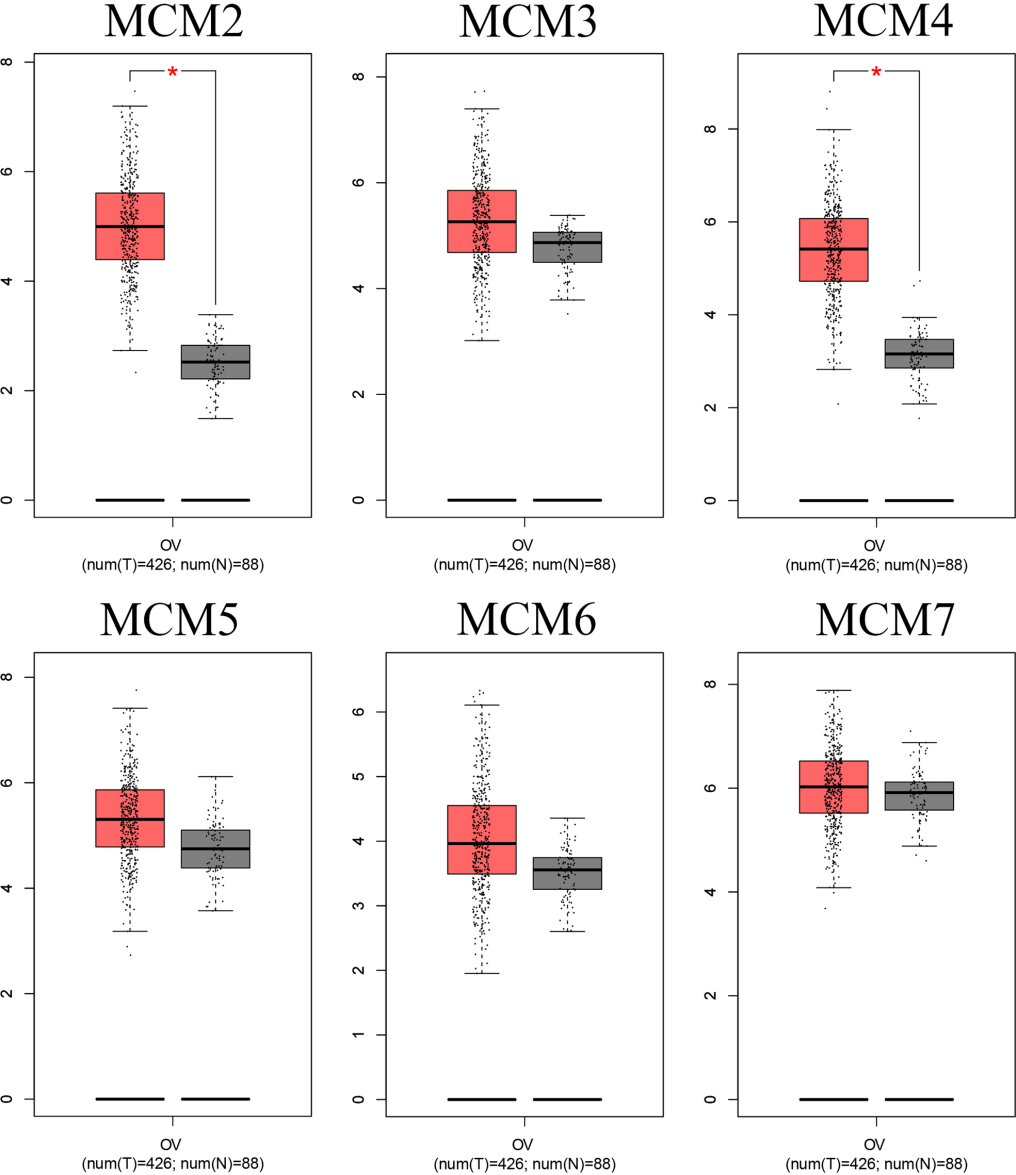
mRNA level of the MCMs in OC. The mRNA level of the MCM complex in OC compared to that in normal ovary tissues based on the GEPIA database. *p < 0.05.

**Figure 4:**
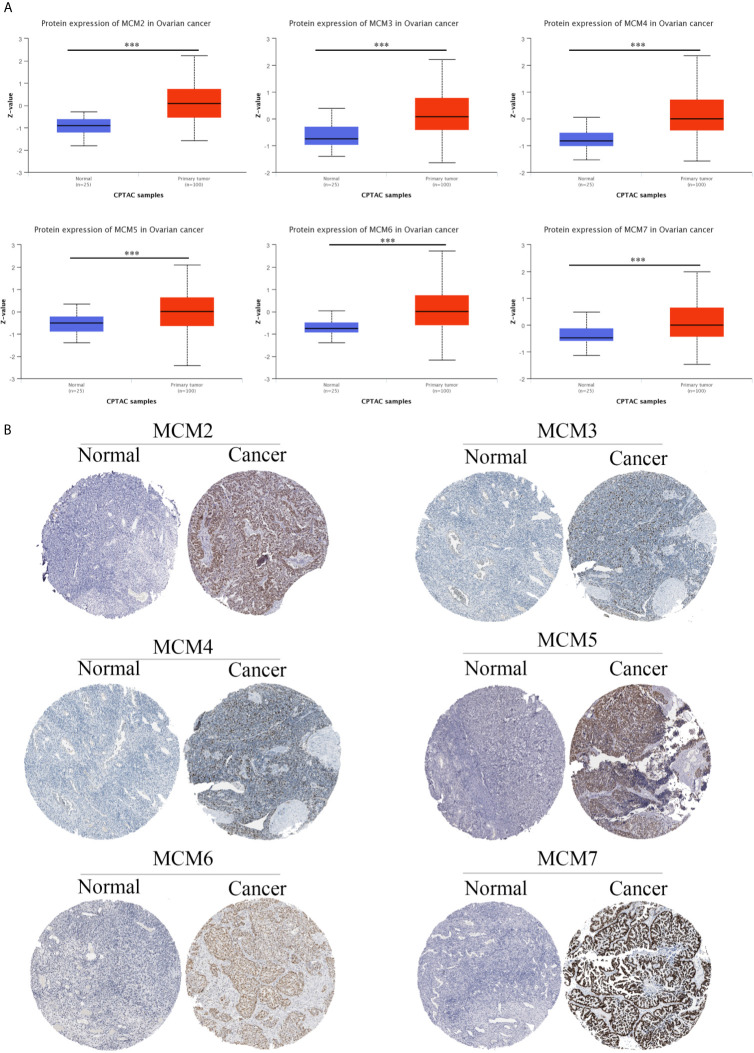
MCM protein expression in OC. The protein expression of the MCM complex in OC based on the CPTAC database **(A)** and HPA database **(B)** ***p < 0.001.

Interestingly, we found that the MCM complex members were all enhanced in the ovarian cancer dataset. Analyses of eight unique datasets were performed for MCM2, MCM4, and MCM6 in ovarian cancer, and three were significant. For MCM3 and MCM5, there were eight unique datasets in OC, and one of them was statistically significant. MCM7 had eight unique datasets in OC, and four datasets were significant, which indicated that the MCM complex had significant and positive correlations with the formation, development, and progression of OC (**Figure 2**). Therefore, we further detected the transcriptional level of the MCM complex in OC tissues compared to that in normal ovary tissues based on the GEPIA database (**Figure 3**). The RNA levels of MCM2 and MCM4 were significantly increased in OC samples compared to those in normal ovary samples. We also extracted protein level data from the CPTAC dataset, which showed that the expression of MCM2–7 was significantly increased in OC tissues compared to that in normal ovary tissues (**Figure 4A**). Furthermore, the HPA database also indicated that the protein expression of MCM2–7 was obviously increased in OC tissues and that these MCM complexes were primarily located in the cell nucleus (**Figure 4B**). In summary, these results indicate that ectopic expression of MCM2–7 is a significant feature in OC patients and may be used to diagnose OC patients.

### Possible Regulatory Mechanisms of the MCM Complex in OC Patients

**Figure 5:**
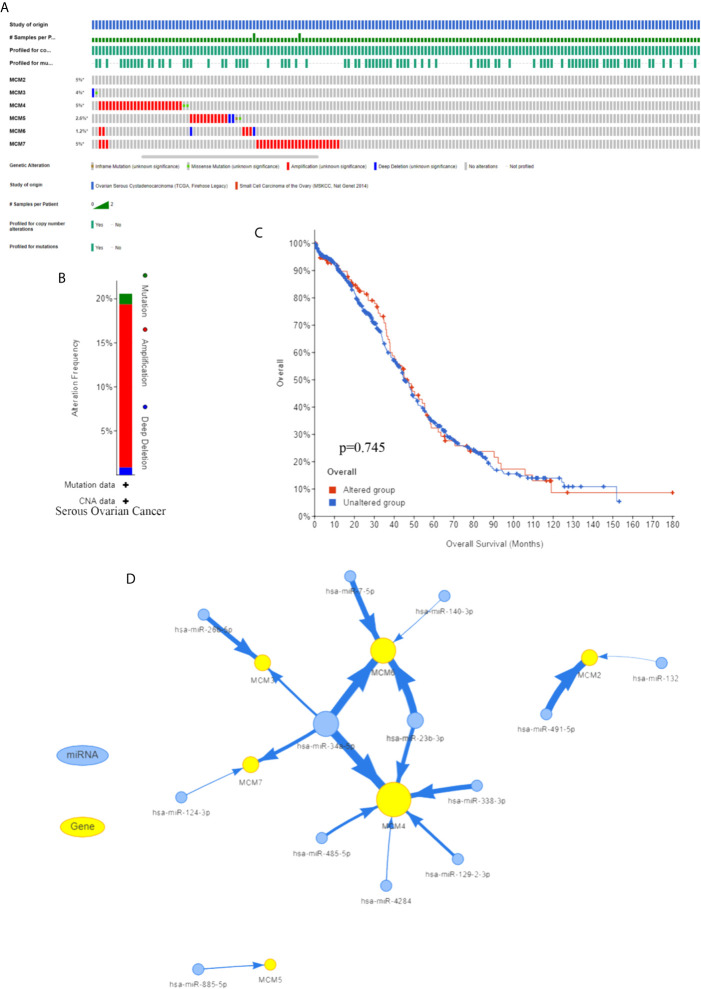
Regulation of the MCMs **(A)** Frequency of the MCM complex based on the cBioProtal database. **(B)** MCM gene alterations in serous ovarian cancer. **(C)** Kaplan–Meier plots of the OS of OC patients with or without MCM alterations. **(D)** The network among MCMs and miRNAs.

To further explore the possible regulatory mechanisms of the MCM complex in OC progression, upstream regulators were progressively explored. First, we extracted data on MCM complex alterations in OC from the cBioProtal database. The serous ovarian cancer dataset indicated that the percentages of DNA alterations of MCMs were 5% (MCM2), 4% (MCM3), 5% (MCM4), 2.6% (MCM5), 1.2% (MCM6), and 5% (MCM7) (**Figures 5A, B** **)**. Next, we analyzed the correlation between MCM complex alterations and survival outcome. However, we found that alterations in the MCM complex were not correlated with OC patient prognosis (**Figure 5C**). These results indicated that dysregulation of the MCM complex was not primarily attributed to DNA alterations.

Subsequently, we found that MCM complex mRNA was regulated by multiple non-coding RNAs, especially hsa-miR-34a-5p and hsa-miR-23b-3p, which indicated that regulation of non-coding RNA might play a key role in alterations of the MCM complex (**Figure 5D**). Moreover, A2780 cells were transfected with hsa-miR-34a-5p or hsa-miR-23b-3p mimics, which significantly increased the level of hsa-miR-34a-5p or hsa-miR-23b-3p, respectively (**Figure S1A**). The qPCR analysis showed that the levels of MCM3, MCM4, MCM6, and MCM7 were significantly enhanced by hsa-miR-34a-5p mimics, and the levels of MCM4 and MCM6 were markedly increased by hsa-miR-23b-3p (**Figure S1B**). A previous study showed that hsa-miR-34a-5p was decreased in OC samples (31). Another study indicated that hsa-miR-23b-3p expression was decreased in endometrioid endometrial carcinoma (EEC) at grades 3 compared to grades 1 (32). These results indicated that the downregulation of hsa-miR-34a-5p or hsa-miR-23b-3p can increase the expression of MCMs to promote OC progression.

**Figure 6:**
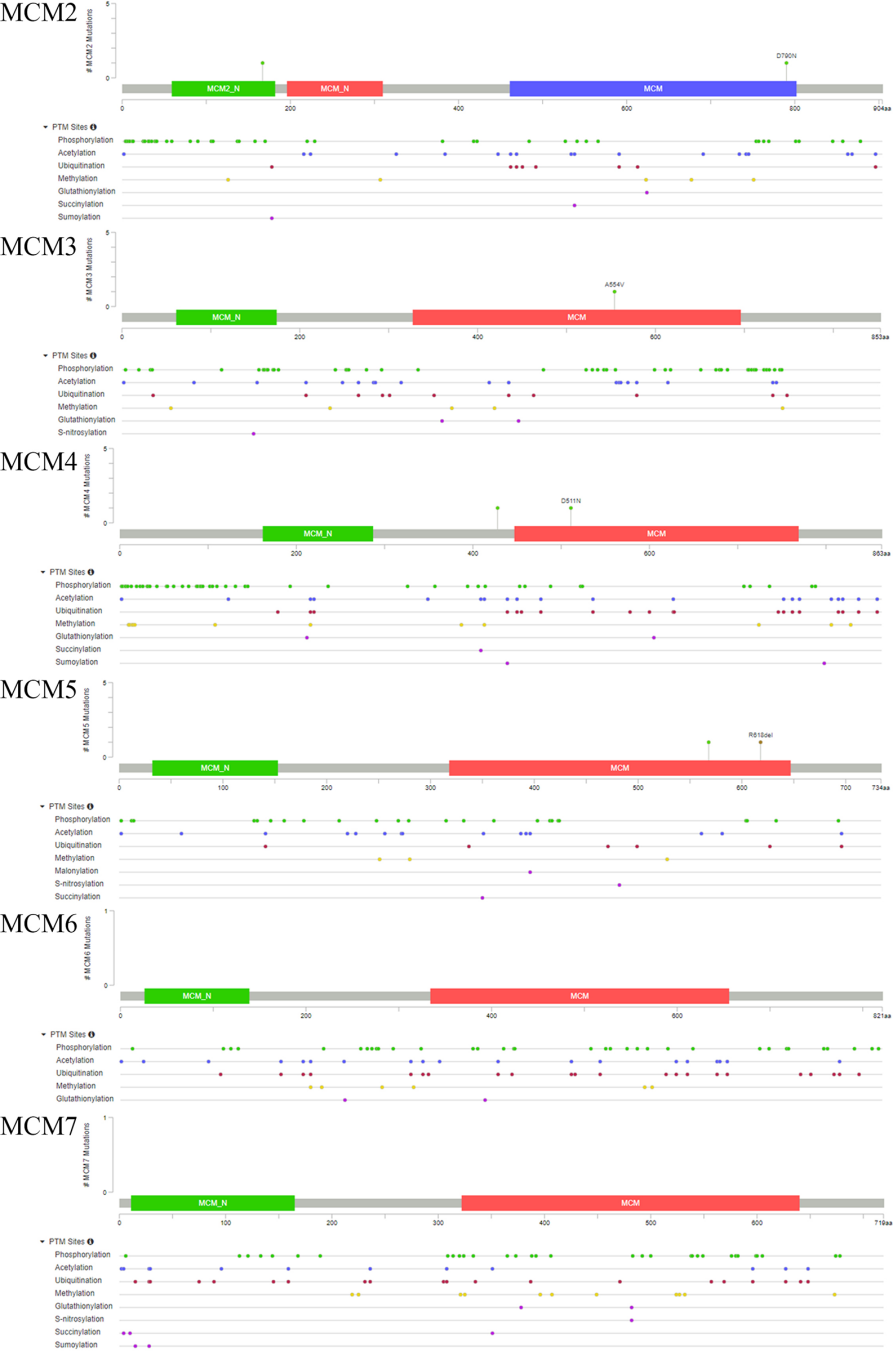
Protein secondary structures of MCM2–7.

**Figure 7:**
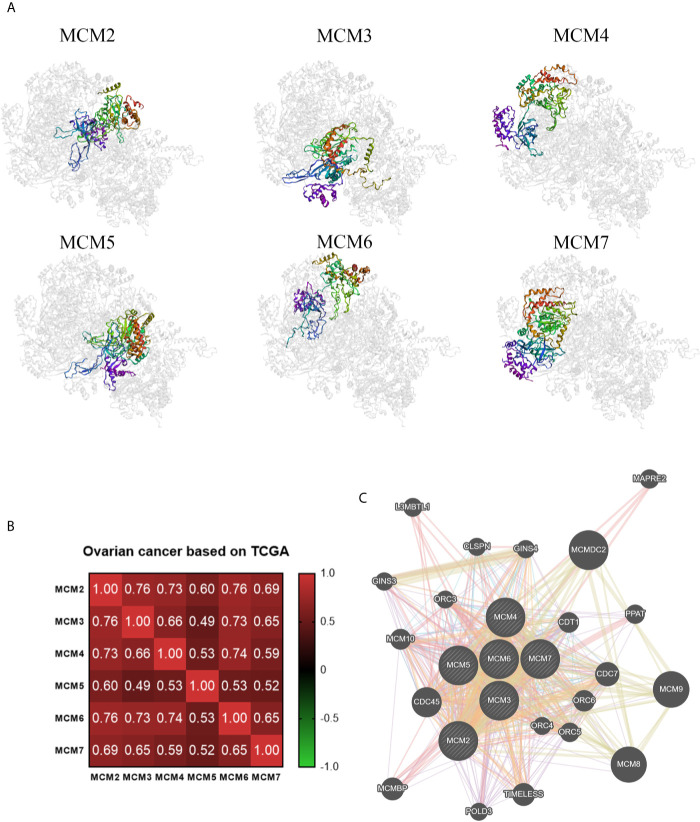
Coexpression and interactions with MCM complex. **(A)** The tertiary structure of MCMs based on the PDB database. **(B)** Spearman’s correlation analysis of the MCMs. **(C)** The protein–protein interaction network among the MCM members based on the GeneMANIA dataset.

Ultimately, we also analyzed the secondary and tertiary structures of the MCM complex based on the PDB database. We found that these DNA regulators possessed domains similar to MCM and MCM_N, which suggested that these MCMs might have similar functions or could combine with each other (**Figure 6**). The secondary structure of the members of the MCM complex also suggested that they had different sites for chemical modifications, such as phosphorylation, acetylation, ubiquitination, methylation, glutathionylation, succinylation, sumoylation, and S-nitrosylation. Moreover, we also extracted the available data of the protein phosphorylation of MCM proteins from the CPTAC database, including MCM2, MCM3, MCM4, and MCM6 (**Figure S2**), which showed that the phosphorylation levels of MCM2, MCM4, and MCM6 were significantly increased in OC samples compared to those in normal ovary samples. These results indicated that post-transcriptional protein modifications might be involved in the activation of these MCMs. Then, the three-dimensional structure of the MCMs was modeled using the PDB database (**Figure 7A**). We found that they could interact to form a hexamer, which agreed with the secondary structure results. Furthermore, Spearman’s correlation analysis indicated a significantly positive correlation between the levels MCM2/3/4/5/6/7 and those of other MCMs (**Figure 7B**), which indicated that the expression of MCMs follows a similar pattern as a form of positive feedback regulation.

### Function Enrichment of the MCM Complex in OC

**Figure 8:**
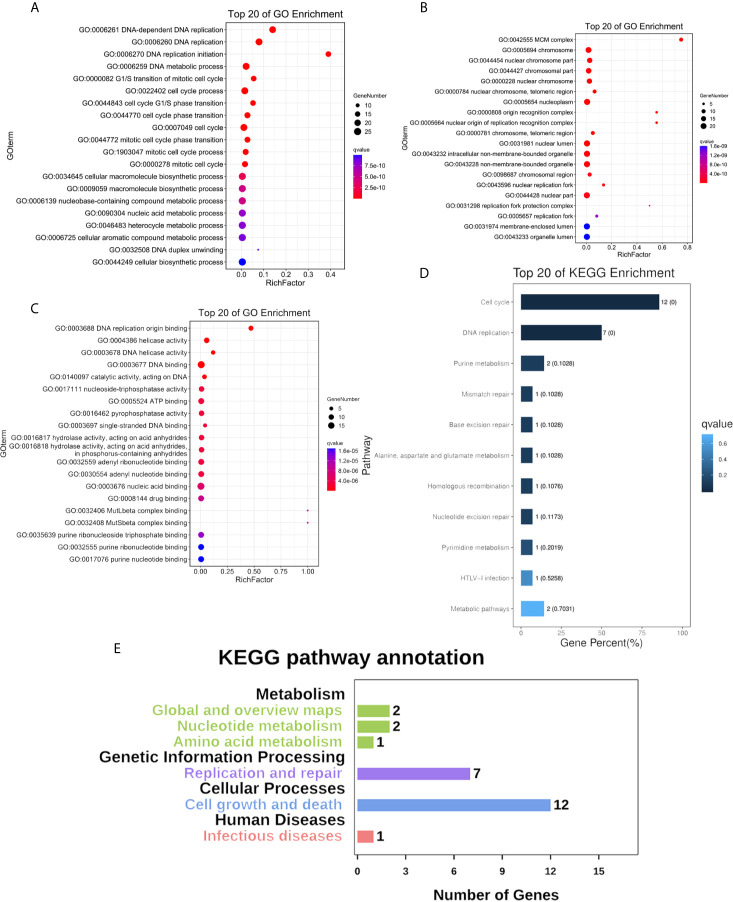
Functional enrichment of the MCM complex and neighbouring interaction genes in OC patients. **(A)** Biological progression terms. **(B)** Cellular component terms. **(C)** Molecular function terms. **(D)** KEGG terms. **(E)** KEGG annotation.

**Figure 9:**
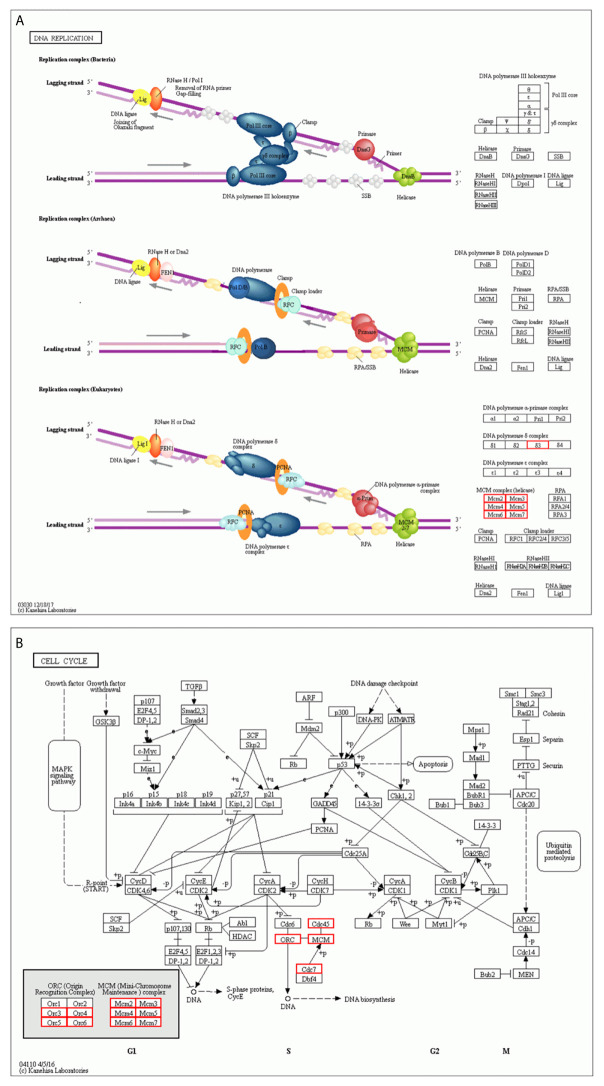
KEGG pathway enrichment analysis **(A)** DNA replication terms based on the MCM complex and neighboring interaction genes. **(B)** Cell cycle terms based on the MCM complex and neighboring interaction genes.

We constructed a network for the MCM complex, which included 20 other genes, using GeneMANIA (**Figure 7C**). The MCM complex could interact with these genes, such as L3 MBTL1, CLSPN, GINS4, GINS3, CDC7, CDC45, POLD3, TIMELESS, ORC3, ORC4, ORC5, ORC6, CDT1, PPAT, MAPRE2, MCM8, MCM9, MCM10, MCMDC2, and MCMBP. We extracted GO and KEGG pathway data for these genes from the DAVID database. In the terms of biological progression, these genes were enriched in DNA-dependent DNA replication, DNA replication, DNA replication initiation, DNA metabolic process, and cell cycle process, indicating that they play a key role in DNA replication and the cell cycle (**Figure 8A**). In terms of cell components, these genes were enriched in the MCM complex, chromosome, nucleoplasm, nuclear origin of replication recognition complex, and nuclear lumen, indicating that they are primarily located in the cell nucleus and participate in the composition of the nucleus (**Figure 8B**). In terms of molecular function, these genes were enriched in DNA replication origin binding, helicase activity, ATP binding, pyrophosphatase activity, and drug binding, indicating that that the function of these genes mainly involves DNA replication and energy metabolism (**Figure 8C**). From the KEGG analysis, we found that these genes were mainly enriched in the cell cycle, DNA replication, purine metabolism, mismatch repair, and metabolic pathways (**Figure 8D**). Furthermore, the KEGG pathway analysis indicated that these genes were involved in nucleotide metabolism, amino acid metabolism for metabolism terms, replication and repair for genetic information processing terms, and cell growth and death for cellular processes (**Figure 8E**). Furthermore, we extracted KEGG signaling pathway maps for DNA replication (**Figure 9A**) and the cell cycle (**Figure 9B**), which indicated the role of the MCM complex in the progression of DNA replication and the cell cycle. Taken together, these results indicated that the MCM complex might promote DNA replication and accelerate the cell cycle by directly activating DNA replication, promoting DNA biosynthesis, and strengthening cell metabolism.

### Association of the MCM Levels With Immune Infiltration in OC

**Figure 10:**
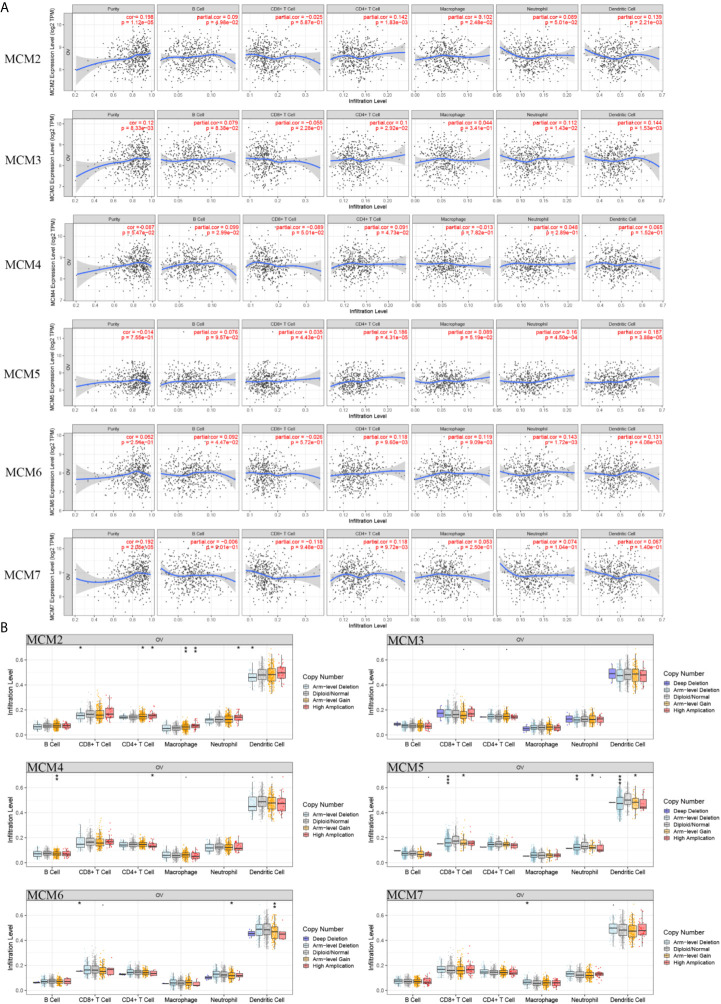
Immune infiltration of the MCMs (Log Ratio > 10). **(A)** Cancer purity and immune infiltration. **(B)** CNV affecting the distribution based on the TIMER database. *p < 0.05, **p < 0.01, ***p < 0.001.

Subsequently, we confirmed the correlation between the mRNA level of MCMs and immune infiltration levels in OC based on the TIMER database. These results indicated that the MCM2, MCM3, MCM4, and MCM7 mRNA levels were closely related to tumor purity (**Figure 10A**). The levels of MCM2, MCM4, and MCM6 were closely correlated with B cells. Only MCM7 was correlated with CD8+ T cells. Furthermore, we found that all the MCMs were significantly correlated with CD4+ T cells. MCM2 and MCM6 were correlated with macrophages. MCM3, MCM5, and MCM6 were closely correlated with neutrophil infiltration. MCM2, MCM3, MCM5, and MCM6 were obviously related to dendritic cells.

Furthermore, the CNV of MCM2 had a significant correlation with the infiltrating levels of CD8+ T cells, CD4+ T cells, macrophages, neutrophils, and dendritic cells. The CNV of MCM4 was correlated with B cells and CD4+ T cells. The MCM5 and MCM6 CNV levels were significantly associated with CD8+ T cells, neutrophils, and dendritic cells. The CNV of MCM7 was only significantly correlated with macrophages (**Figure 10B**).

### Prognostic Value of MCM Complex Members in OC Patients

**Figure 11:**
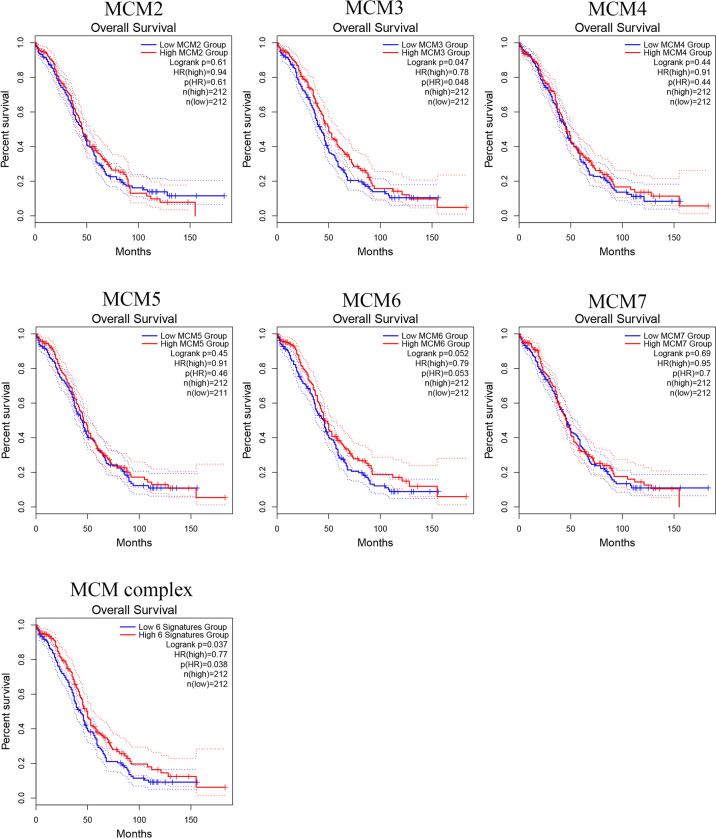
Prognostic value of the MCM complex members in OC. Survival analysis of the MCM2, MCM3, MCM4, MCM5, MCM6, and MCM7 signatures and the six-gene MCM signature in OC patients based on the GEPIA database.

To further test whether the MCM complex signatures have independent prognostic value in OC patients, we extracted MCM level data and prognostic from TCGA, as shown in **Figure 11**. However, we found that the single MCMs did not have prognostic value, but when we tested the prognostic value of the six-gene signature for OC, we found that it was significantly and negatively correlated with OC patient prognosis (log-rank p = 0.037). These results indicated that the six-gene MCM complex signature might be a good prognostic signature for OC patients.

### Verification of the Drug Sensitivity of the MCM Complex

Furthermore, we utilized GSCALite (http://bioinfo.life.hust.edu.cn/web/GSCALite/) to analyze the drug sensitivity of the MCM complex in OC. We found that the MCMs were significantly correlated with chemotherapy resistance based on the GSCALite database (**Figures S3**, **S4**). These results suggest that the MCM complex is involved in multidrug resistance in OC patients.

## Discussion

Many studies have indicated that the MCM complex is involved in DNA replication progression, molecular signaling pathways, and the cell cycle (4). Dysregulation of the MCM complex has been found in multiple cancer types, especially gastric cancer (33), liver cancer (34), cervical cancer (7), prostate cancer (35), colon cancer (13), clear-cell renal cell carcinoma (36), glioma (18), and ovarian cancer (37). However, there is no comprehensive analysis of the MCM complex in OC in the literature. Hence, we systematically analyzed the mRNA and protein levels, protein structure, protein interactions, functional enrichment, immune infiltration, and prognostic value of the MCM complex.

This study indicated that the MCM2 and MCM4 mRNA levels were significantly increased in OC tissue compared to those in normal ovary tissue based on TCGA and the GTEx databases, whereas, the mRNA levels of MCM3, MCM5, MCM6, and MCM7 were not significantly increased. Liu et al. found that MCM2 could be a potential biomarker for the prognosis and progression of OC (38). Moreover, MCM2 knockdown could increase the carboplatin sensitivity of OC cells (39). Paclitaxel and eribulin could decelerate the cell cycle by inhibiting the level of MCM4 mRNA (40). In our study, we found that the protein expression of the MCM complex was significantly increased in OC tissue compared to that in normal ovary tissue based on the CPTAC and HPA databases. Interestingly, only the mRNA levels of MCM2 and MCM4 were increased, while the protein levels of all MCMs were increased, which may be caused by multiple post-transcriptional regulatory mechanisms, such as the regulation of non-coding RNA. Chuang et al. also found that inhibition of one MCM could attenuate the levels of all the other MCMs in mammalian cells (41), which indicated that high levels of MCM2 and MCM4 could further upregulate the expression of other MCMs in OC. Issac MSM and his colleagues indicated that the expression of MCM2, MCM4, and MCM6 was obviously increased in breast cancer compared to that in corresponding para-cancerous tissues (42). High expression of MCM3, an independent biomarker, was found to be correlated with poor prognosis (43). Another study also indicated that MCM5 was a novel biomarker for the diagnosis of OC (44). Ota T et al. suggested that MCM7 had potential prognostic value in OC (45). These results and those of our study indicate that expression of the MCM complex proteins is correlated with poor prognosis in OC.

DNA mutation is a driver event in cancers (46). We also explored the correlation between DNA alterations and the MCM complex, but we found that the frequency of DNA alterations was not high, and it was unlikely that DNA alterations led to dysregulation of the MCM complex. Non-coding RNA is a clear example of how inherited epigenetic changes can play a role in carcinogenesis, and non-coding RNA is the most abundant type of RNA (47). Therefore, we detected the potential regulatory association among miRNAs and the MCM complex. Our results indicated that multiple miRNAs could interact with the MCM complex, especially miR-34a-5p and miR-23b-3p. Zuo Y et al. found that miR-34a-5p was decreased in OC cells and that this miRNA could repress proliferation, induce cell cycle arrest at the G1 phase, and enhance apoptosis levels (48). miR-23b-3p was found to have a biological role in reducing proliferation, migration, and invasion in cervical cancer cells (49). However, the role of miR-23b-3p in OC is still unclear. These results in combination with our results suggest the regulation of non-coding RNA is correlated with disorder of the MCM complex.

Post-translational modifications can regulate protein–protein interactions, protein stability and protein localization (50). The function and activity of proteins are mediated by multiple post-translational modifications, including phosphorylation, acetylation, ubiquitination, methylation, glycosylation, glutathionylation, succinylation, sumoylation, and S-nitrosylation (51). We found that MCMs had multiple modification sites, which could be subjected to post-translational modifications. These results indicated that targeting protein modifications can alter the expression of these MCMs to impede the development and progression of OC. Inhibition of cancer-promoting kinases has been considered to be an established therapeutic strategy for treating many cancer types (52). In our previous study, FGFR tyrosine kinase inhibitors (TKIs) were shown to repress the proliferation, differentiation, and migration of cancer cells, showing that they have great potential for development in precision medicine and individualized treatment of cancer patients (53). Therefore, further research on the development of post-translational modifications and, in particular, kinase inhibitors targeting the MCM complex would be beneficial for cancer treatment. Meagher M and colleagues found that MCM2–7 formed a hexamer to power DNA strand separation of the replication forks of eukaryotes and archaea (54). Our three-dimensional structure results of these MCMs also indicated that they interact with each other to form a hexamer, resulting in accelerated DNA replication. Hence, hindering assembly of the MCM complex might also be a potential direction for treating OC.

Moreover, our study indicated that there was a positive association among the MCMs in OC according to TCGA. We also constructed a PPI network of the MCM complex, which revealed that the MCM complex had strong connections with L3MBTL1, CLSPN, GINS4, GINS3, CDC7, CDC45, POLD3, TIMELESS, ORC3, ORC4, ORC5, ORC6, CDT1, PPAT, MAPRE2, MCM8, MCM9, MCM10, MCMDC2, and MCMBP. This network could be used to predict the biological function and molecular signaling pathway of the MCM complex *via* GO and KEGG enrichment analyses. In our study, we found that the MCM complex was involved in DNA replication, the cell cycle, infectious diseases, and metabolic pathways. In a previous study, instability of the MCM complex was found to destabilize the interaction between the MCM complex and DNA (55). The MCM complex is a licensing regulator of DNA replication and promotes DNA synthesis to accelerate the cell cycle (56). There is no study available that has investigated the function of the MCM complex in metabolism, but purine nucleotide metabolism is crucial for DNA replication (57), which suggests a potential effect of MCMs on purine nucleotide metabolism in carcinogenesis. Two previous studies indicated that MCM6 was involved in immune progression dysregulation and might be a target for immunotherapy for systemic lupus erythematosus (58) and anaplastic oligodendroglioma (59). Although these studies are based on immunological diseases, they suggest a number of possible ideas for future cancer research. In our study, we found a correlation between the MCM complex and immune infiltration in OC. The expression of MCM2, MCM4, MCM5, MCM6, and MCM7 was closely correlated with six main types of immune cells (B cells, CD8+ T cells, CD4+ T cells, macrophages, neutrophils and dendritic cells). These results indicated that the MCM complex might play a key role in the immune status of OC patients.

Finally, our study found that MCM2–7 could not be used as single markers for the diagnosis and prognosis of OC, but the six-gene prognostic signature (MCM2–7) could be used as a good biomarker for OC patients. Raunak Shrestha et al. found that the expression of the MCM protein complex was a potential treatment target in MEK inhibitor (MEKi)-resistant OC cell lines (60). We also confirmed the drug sensitivity of the MCM complex in OC based on the GSCALite database, which indicated that the MCMs had a close correlation with chemotherapy resistance in the development and progression of OC. These results both indicated the significance of the MCMs in the drug sensitivity of OC patients.

Clearly, this study has some limitations. First, all the data are from public databases. Further experiments are needed to validate these results *in vivo* and *in vitro*. Subsequently, information on the histopathologic type of OC is not available in many public databases, which is of limited help in understanding the specific role of different MCMs in different ovarian cancers. Additionally, the molecular mechanisms of the role of the MCM complex in OC should be further explored. Ultimately, this study, as a retrospective study, requires further study to support its results.

## Conclusion

In our study, we comprehensively analyzed the role of the MCM complex in OC, including the transcriptional and post-transcriptional levels, genetic alterations, coexpression, PPIs, protein structure, immune infiltration, and prognostic values. In summary, the results of this study indicate that MCMs, as oncogenes, promote the development and progression of OC by activating DNA replication, accelerating the cell cycle, and influencing the immune response.

## Data Availability Statement

The original contributions presented in the study are included in the article/**Supplementary Material**. Further inquiries can be directed to the corresponding authors.

## Author Contributions

YL, JZ, and QZ analyzed the data. FQ, XZ, and LC used online tools. YL, JL, and DW designed the project, selected the analyzed results, and wrote the paper. All authors contributed to the article and approved the submitted version.

## FUNDING

The present study was supported by the Scientific Research Program of Hunan Provincial Health Commission (Grant No. B2019115), and the Natural Science Foundation of HuNan Province, Hunan Provincial Technology Innovation Guidance Plan Clinical Medical technology innovation guidance project (2018SK51510).

## Conflict of Interest

The authors declare that the research was conducted in the absence of any commercial or financial relationships that could be construed as a potential conflict of interest.

## Supplementary Material

Supplementary Figure 1
**(A)** Expression of miR-23b-3p and miR-34a-5p in A2780 cells transfected with miR-23b-3p mimics or miR-34a-5p mimics was evaluated by RT−qPCR, respectively. **(B)** Expression of MCM2, MCM3, MCM4, MCM5, MCM6, and MCM7 in A2780 cells transfected with miR-23b-3p mimics or miR-34a-5p mimics was evaluated by RT−qPCR, respectively.

Supplementary Figure 2 Protein phosphorylation analysis using CPTAC database for MCMs.

Supplementary Figure 3 CTRP drug sensitivity analysis using the GSCALite database for MCMs.

Supplementary Figure 4 GDSC drug sensitivity analysis using the GSCALite database for MCMs.
