# Supplementary figures and images for: Systemic Analysis of the DNA Replication Regulator MCM Complex in Ovarian Cancer and Its Prognostic Value
Source: Front Oncol. 2021 Jun 9;11:681261. doi: 10.3389/fonc.2021.681261 (PMC8220296; doi:10.3389/fonc.2021.681261)

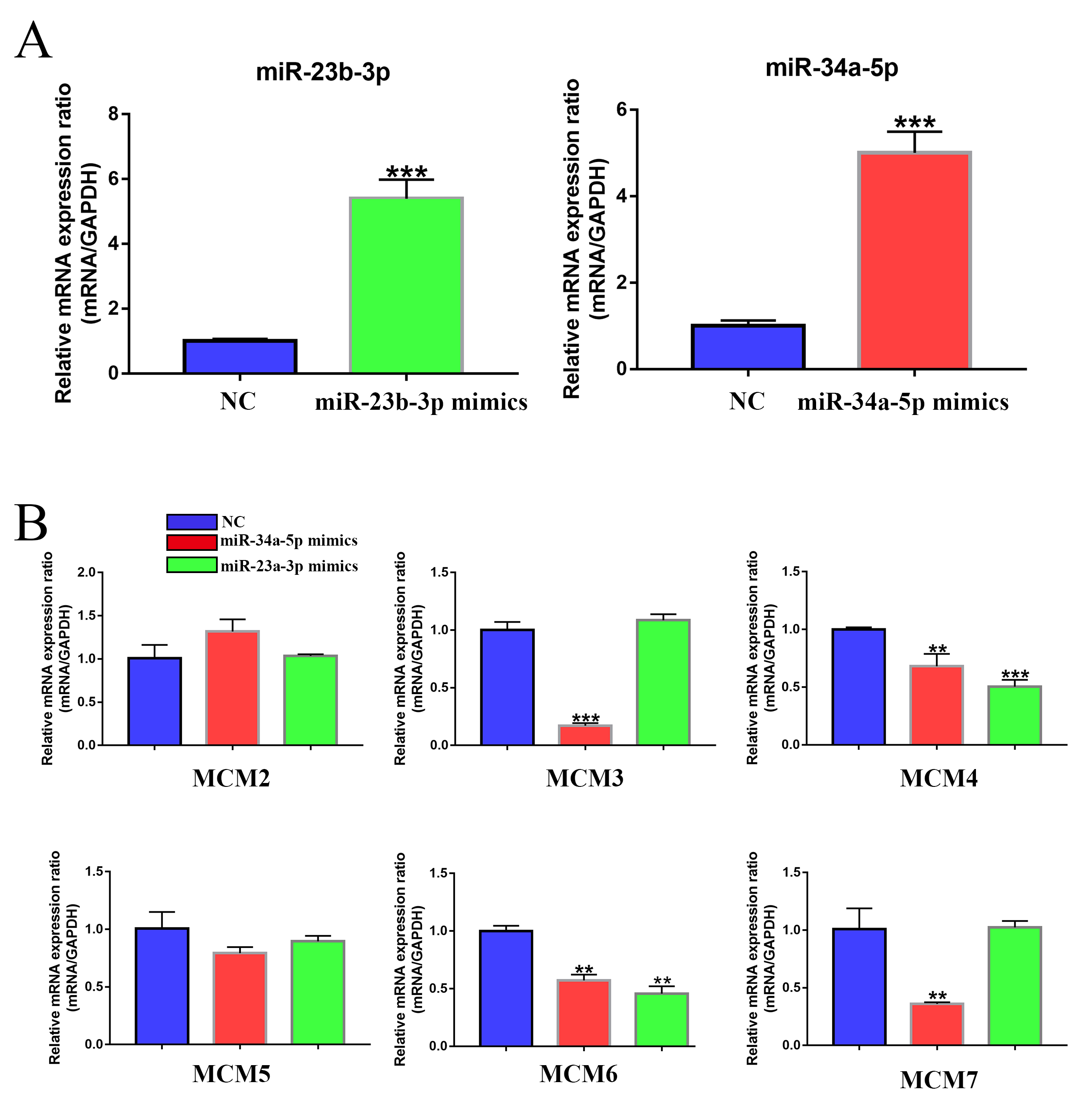

Supplement: Supplementary Figure 1 — (A) Expression of miR-23b-3p and miR-34a-5p in A2780 cells transfected with miR-23b-3p mimics or miR-34a-5p mimics was evaluated by RT−qPCR, respectively. (B) Expression of MCM2, MCM3, MCM4, MCM5, MCM6, and MCM7 in A2780 cells transfected with miR-23b-3p mimics or miR-34a-5p mimics was evaluated by RT−qPCR, respectively. [file Image_1.tif]

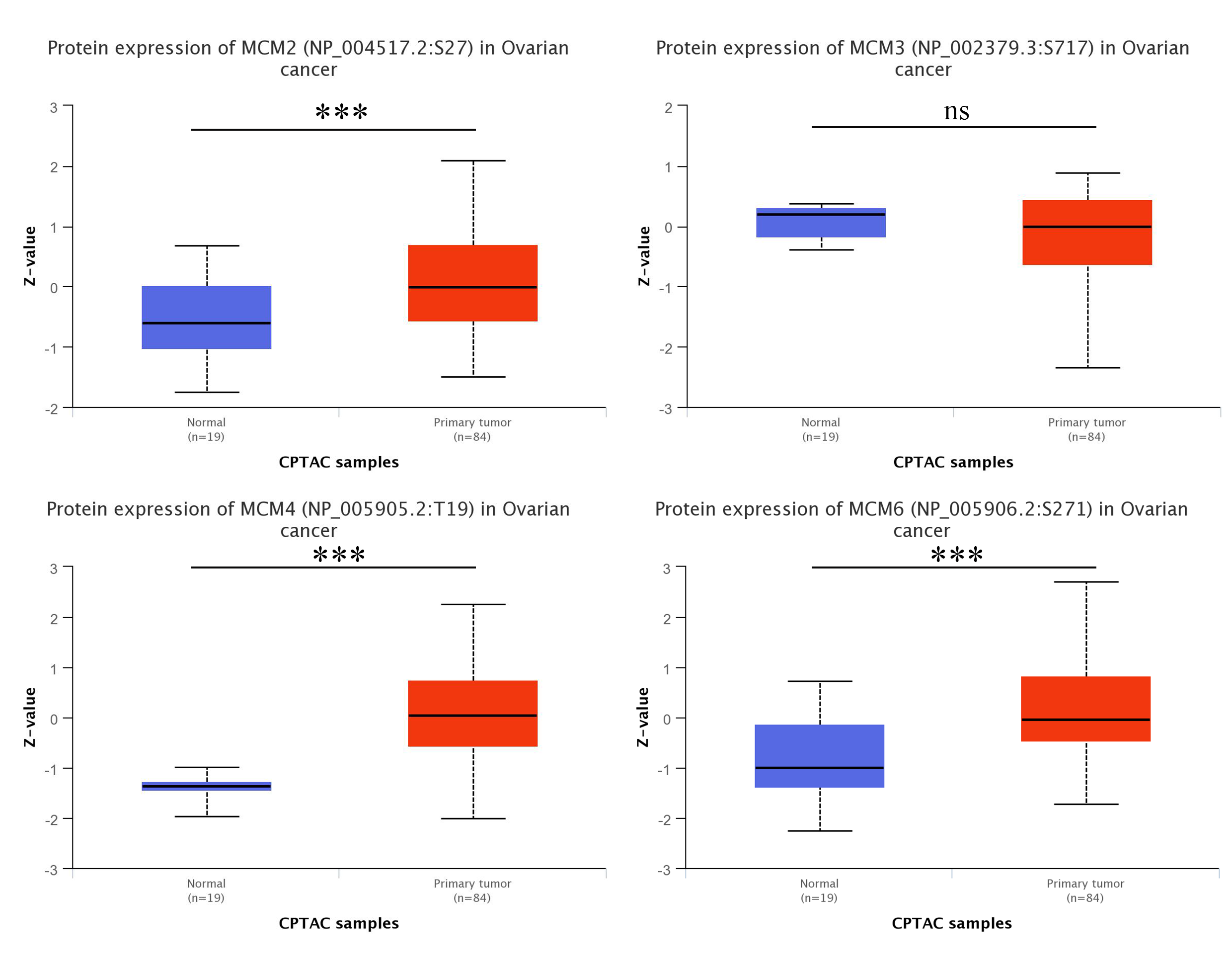

Supplement: Supplementary Figure 2 — Protein phosphorylation analysis using CPTAC database for MCMs. [file Image_2.tif]

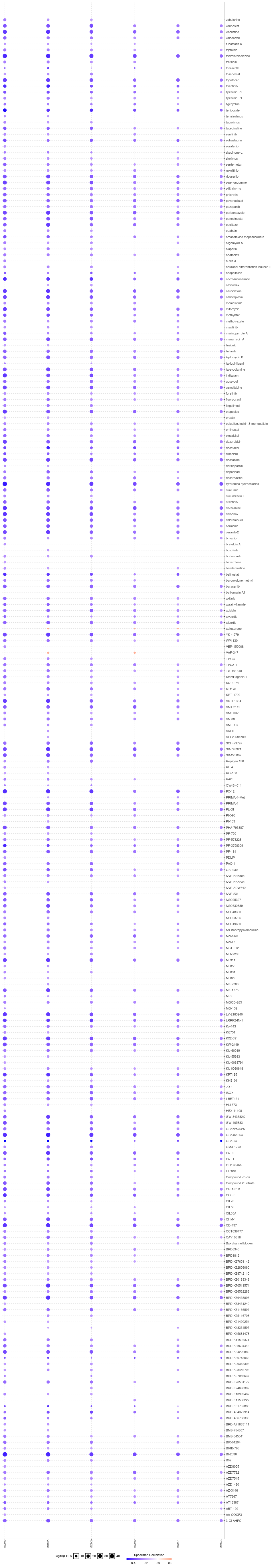

Supplement: Supplementary Figure 3 — CTRP drug sensitivity analysis using the GSCALite database for MCMs. [file Image_3.png]

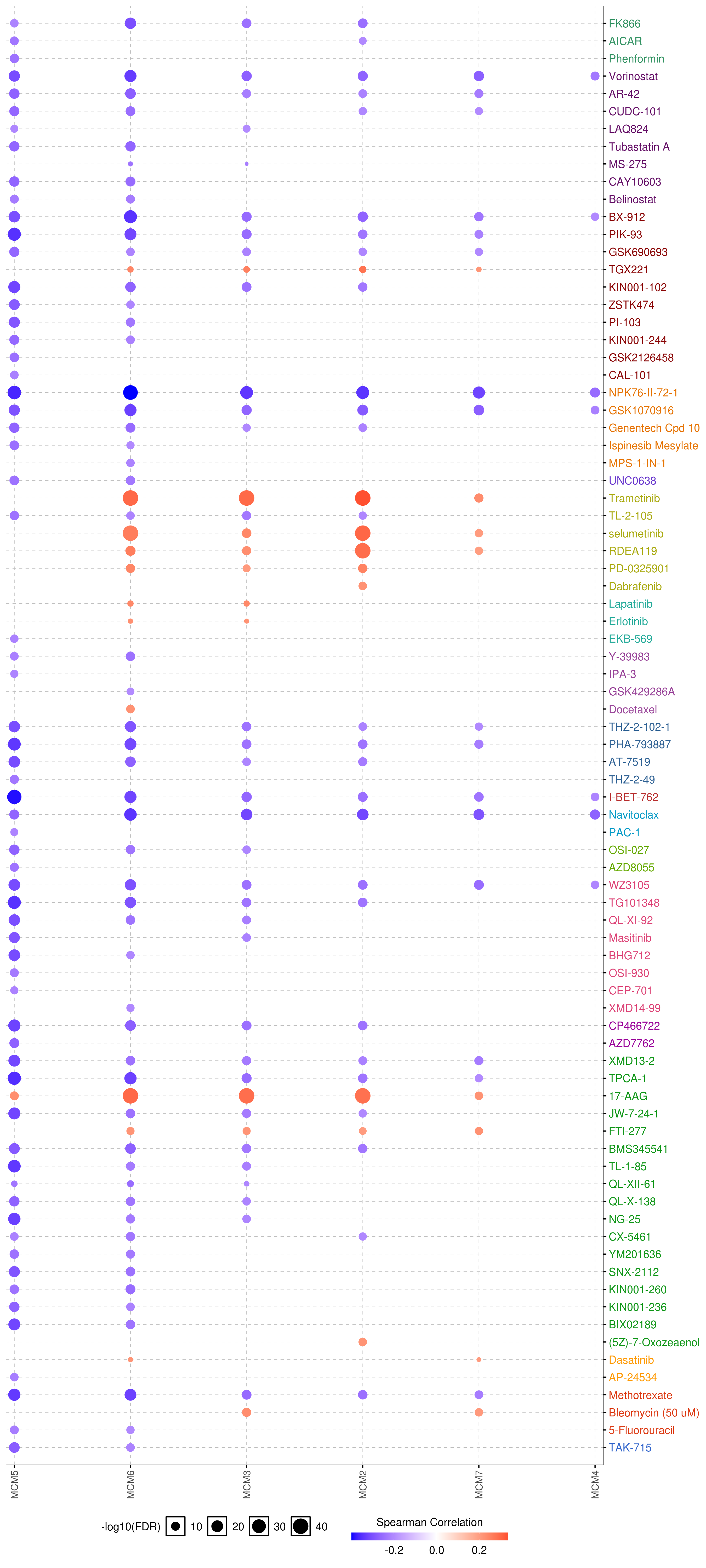

Supplement: Supplementary Figure 4 — GDSC drug sensitivity analysis using the GSCALite database for MCMs. [file Image_4.png]
